# Fatal Outcome Due to Pulmonary Arterial Intramural Hematoma Associated With Stanford Type A Acute Aortic Dissection: A Case Report

**DOI:** 10.7759/cureus.85969

**Published:** 2025-06-13

**Authors:** Yudai Shiwaku, Tatsuya Aonuma, Kanako Matsuda, Takahiro Shiokoshi, Naoki Nakagawa

**Affiliations:** 1 Department of Cardiology, Hokkaido P.W.F.A.C. Engaru-Kosei General Hospital, Engaru, JPN; 2 Division of Cardiology, Nephrology, Pulmonology, and Neurology, Department of Internal Medicine, Asahikawa Medical University, Asahikawa, JPN

**Keywords:** acute chest pain, alveolar hemorrhage, computed tomography, pulmonary arterial intramural hematoma, type a acute aortic dissection

## Abstract

Stanford type A acute aortic dissection (AAD) is associated with a poor prognosis. Pulmonary artery intramural hematoma (PA-IMH) is an underrecognized complication of AAD. Here, we report a case of an 86-year-old man diagnosed with Stanford type A AAD. During emergency transfer to a tertiary care center with cardiovascular surgical capabilities, the patient experienced a cardiac arrest during transport by an ambulance and was subsequently pronounced dead. Postmortem computed tomography (CT) revealed right PA compression and wall thickening consistent with PA-IMH. Additionally, associated ground-glass opacity in the right lower lobe suggested alveolar hemorrhage, likely contributing to circulatory collapse, followed by cardiac arrest and death. We identified two important clinical issues. First, in cases of AAD, PA-IMH should be considered a potential complication when contrast-enhanced CT reveals obstruction or stenosis of the PA. Second, some cases of PA-IMH may present with alveolar hemorrhage, an important prognostic factor of PA-IMH that should be identified using CT with lung window settings.

## Introduction

Stanford type A acute aortic dissection (AAD) is caused by a tear in the media of the ascending aorta and is associated with a poor prognosis. It typically presents with a sudden onset of chest or back pain, followed by a wide range of clinical manifestations resulting from various complications [1,2]. The main complication of Stanford type A AAD is rupture of the ascending aorta [1]. In most cases, the aorta ruptures into the pericardium, leading to cardiac tamponade, with a reported mortality rate of approximately 50% [2]. Pulmonary artery intramural hematoma (PA-IMH) is another fatal complication resulting from rupture due to AAD; however, it remains relatively underrecognized [3]. As PA-IMH typically presents without specific clinical findings, imaging studies, such as computed tomography (CT), are critically important for its diagnosis. PA-IMH is often observed on contrast-enhanced CT as luminal narrowing or occlusion of the PA [4-6]. Here, we report a case of PA-IMH secondary to AAD, complicated by alveolar hemorrhage and a poor clinical outcome.

## Case presentation

**Figure 1:**
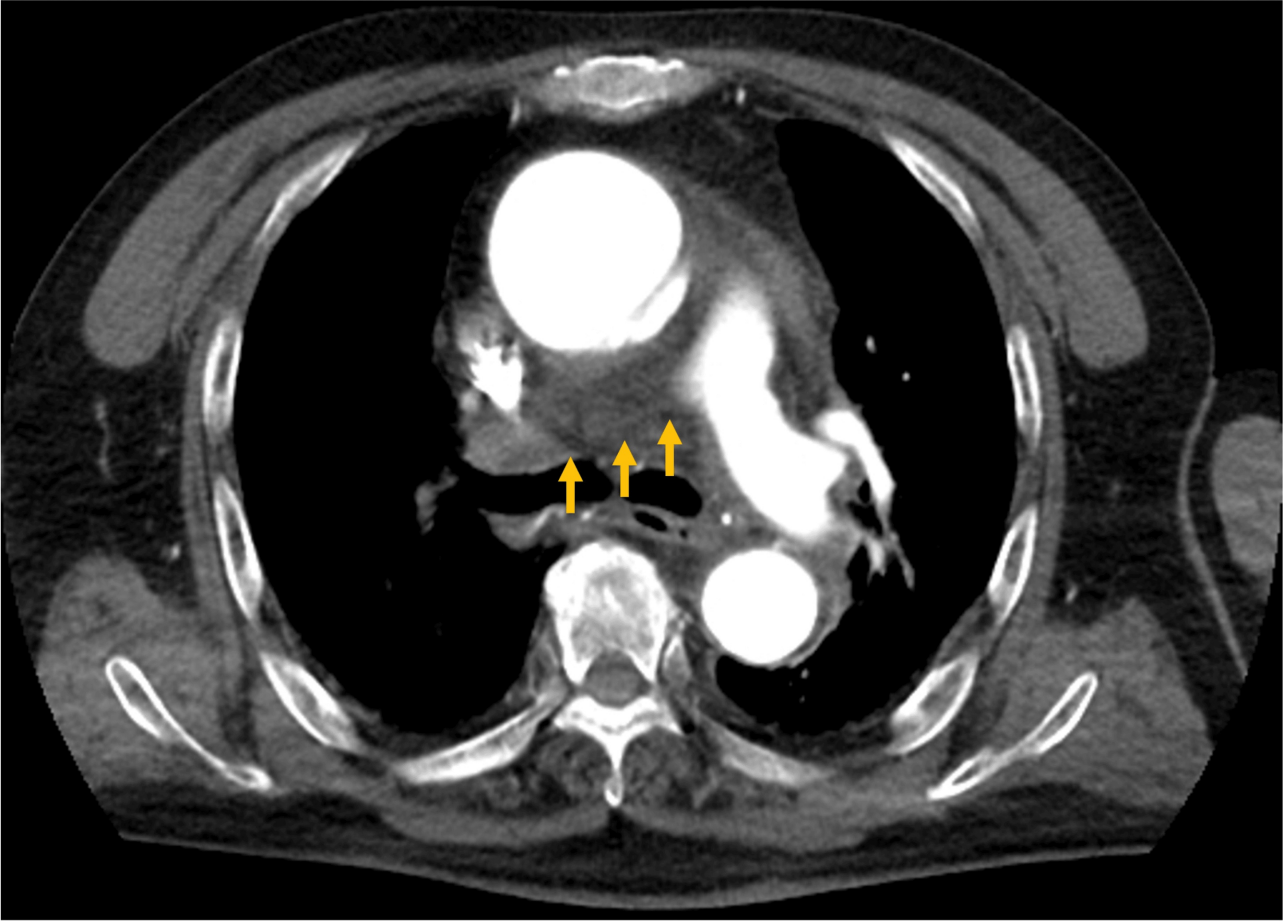
Contrast-enhanced computed tomography showing occlusion of the right pulmonary artery just distal to its bifurcation from the main pulmonary artery (yellow arrow)

An 86-year-old man with hypertension was transported to our hospital at noon with chest pain that began at 6:30 a.m. He had no medical conditions, family history, or smoking history associated with atherosclerotic risk, other than hypertension. On admission, he reported continuous chest pain that had migrated from the epigastric region to the anterior chest. Vital signs on examination were as follows: blood pressure: 127/85 mmHg (right arm) and 106/73 mmHg (left arm); heart rate: 72 beats/min with a regular rhythm; respiratory rate: 20 breaths/min. Supplemental oxygen was initiated during transport due to the development of hypoxemia. On admission, his oxygen saturation was 97% on 3 L/min of supplemental oxygen. Laboratory results showed mildly elevated D-dimer (2.5 ng/mL) and troponin I (84.6 ng/mL) levels. Electrocardiography revealed an incomplete right bundle branch block but no ST-segment or T-wave abnormalities, ruling out acute myocardial infarction. Based on these findings, Stanford type A AAD was strongly suspected. Contrast-enhanced CT confirmed the diagnosis of Stanford type A AAD, with an entry point in the ascending aorta; the dissection was confined to the ascending aorta. The right PA lacked contrast enhancement in the main trunk, raising suspicion of obstruction and possible acute pulmonary thromboembolism (PTE) (Figure 1).

Emergency surgical transfer to a tertiary care center with cardiovascular surgical capabilities was planned. However, the patient developed shock after CT, prompting norepinephrine and aggressive fluid resuscitation. The patient subsequently experienced cardiac arrest during transport by the ambulance and was pronounced dead at 2:00 p.m. after returning to the hospital.

**Figure 2:**
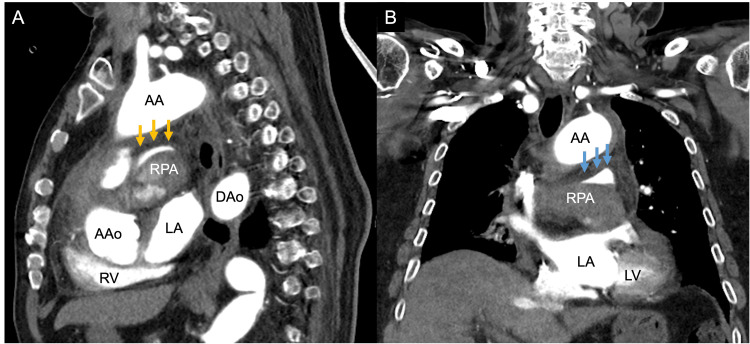
Contrast-enhanced computed tomography of the right pulmonary artery main trunk On sagittal view (A), the right pulmonary artery is compressed from below, resulting in a crescent-shaped lumen (yellow arrow). On coronal view (B), tapering stenosis extends from the main pulmonary artery (blue arrow). AA: aortic arch; AAo: ascending aorta; DAo: descending aorta; LPA: left pulmonary artery; LPV: left pulmonary vein; MPA: main pulmonary artery; RPA: right pulmonary artery

A postmortem contrast-enhanced CT was performed to investigate the cause of death. The sagittal view showed compression of the right PA from below, creating a crescent-shaped lumen, whereas the coronal view showed progressive tapering and narrowing of the vessel (Figure 2).

**Figure 3:**
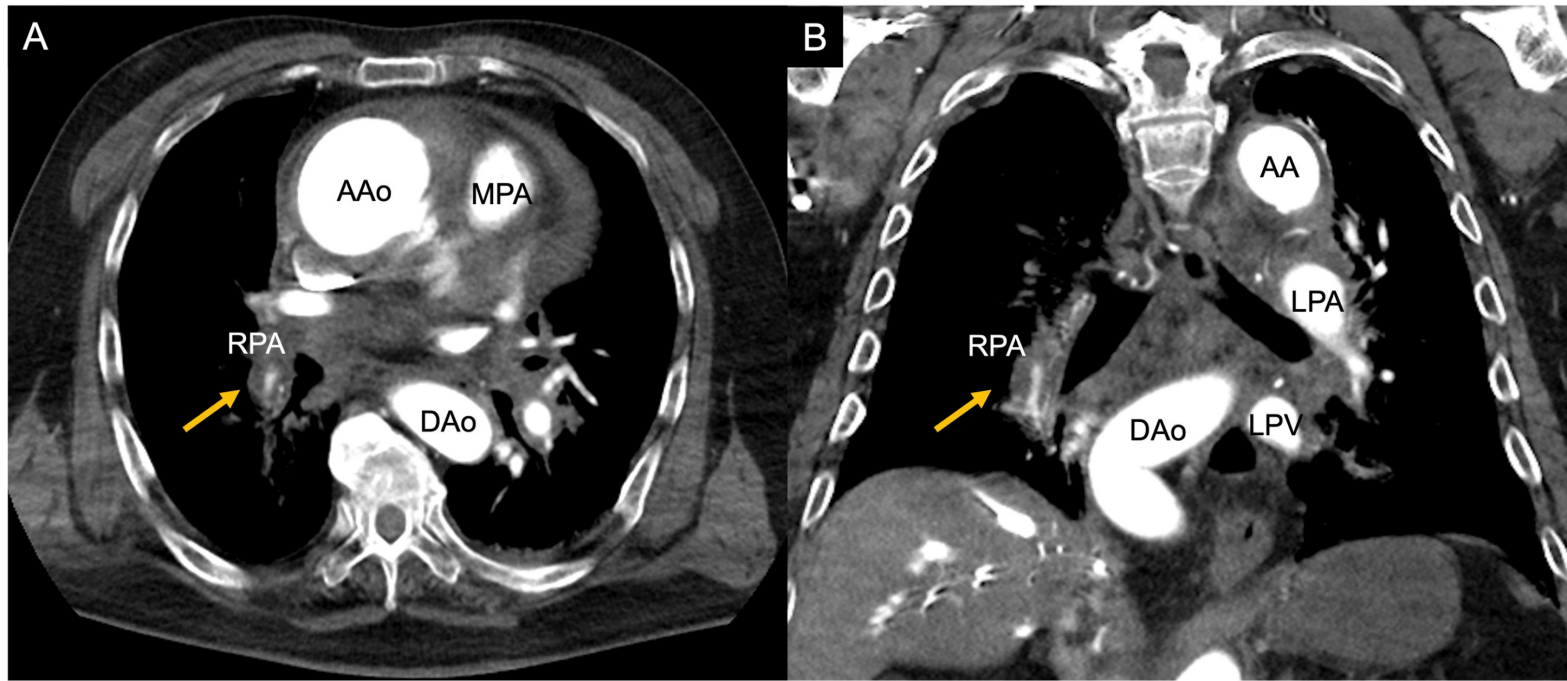
Contrast-enhanced computed tomography of a branch of the right lower lobe pulmonary artery On axial (A) and coronal (B) views, the arterial wall of the right lower lobe pulmonary artery branch is thickened, causing concentric compression of the lumen (yellow arrow). AA: aortic arch; AAo: ascending aorta; DAo: descending aorta; LA: left atrium; LV: left ventricle; RPA: right pulmonary artery

CT also revealed circumferential thickening of the arterial wall and luminal narrowing in a branch of the right lower lobe PA (Figure 3).

**Figure 4:**
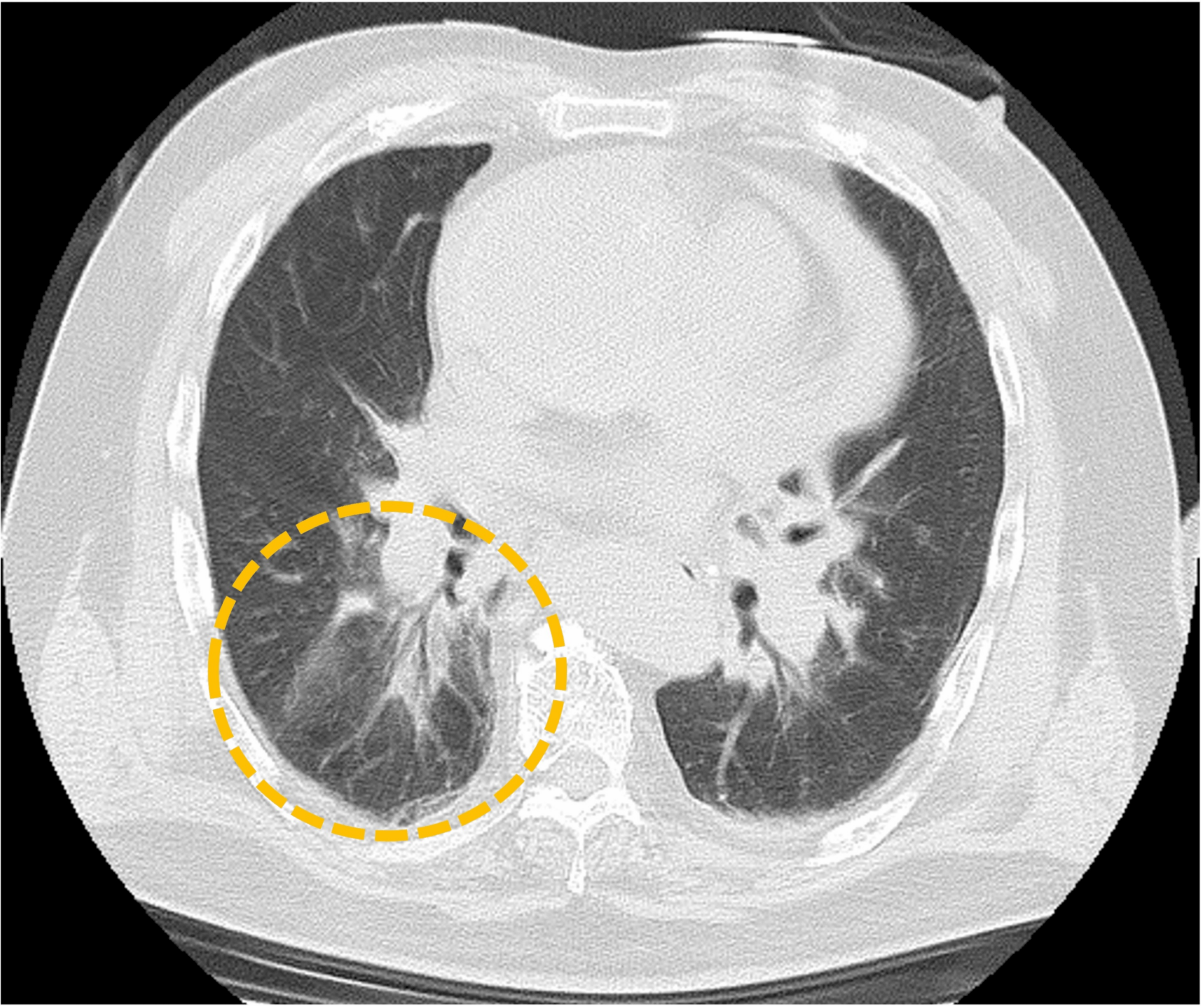
Non-contrast computed tomography in lung window settings In lung window settings, a ground-glass opacity is observed surrounding the hematoma in the right lower lobe pulmonary artery branch, suggesting alveolar hemorrhage (yellow circle).

These findings were inconsistent with PTE. Instead, the pattern, characterized by hematoma extending along the pulmonary arterial wall, was diagnosed as PA-IMH. In addition, lung window settings revealed a localized ground-glass opacity in the right lower lobe (Figure 4).

This region corresponded to the PA branches where the hematoma had extended, suggesting alveolar hemorrhage caused by PA-IMH. Therefore, circulatory insufficiency due to PA occlusion and stenosis, along with alveolar hemorrhage, was considered to have contributed to the patient's clinical deterioration.

## Discussion

This report presents the case of an 86-year-old man with PA-IMH secondary to AAD, complicated by alveolar hemorrhage and a poor clinical outcome. The patient’s clinical course highlighted two important considerations: the need to recognize PA-IMH as a complication of AAD and the prognostic significance of associated alveolar hemorrhage.

First, in cases of AAD, PA-IMH should be considered a potential complication when contrast-enhanced CT reveals obstruction or stenosis of the PA. The ascending aorta and PA trunk share a common adventitia, which caudally becomes the visceral pericardium. During AAD, the media divides into outer and inner layers. Aortic rupture tends to occur at sites where the outer layer is thin. If this thin outer layer forms on the posterior side of the aorta near the PA, rupture may result in hematoma extension into the common adventitia. Because aortic pressure exceeds PA pressure, the hematoma can readily extend along the PA, leading to PA-IMH formation. Although PA-IMH is traditionally considered a rare complication of Stanford type A AAD [4-6], a recent study by Koike et al. reported an incidence as high as 24.7%, suggesting that it may no longer be rare [3]. As CT resolution improves, the number of detectable PA-IMH cases is increasing. PA-IMH should be suspected when the PA lacks enhancement on contrast-enhanced CT in AAD cases.

**Figure 5:**
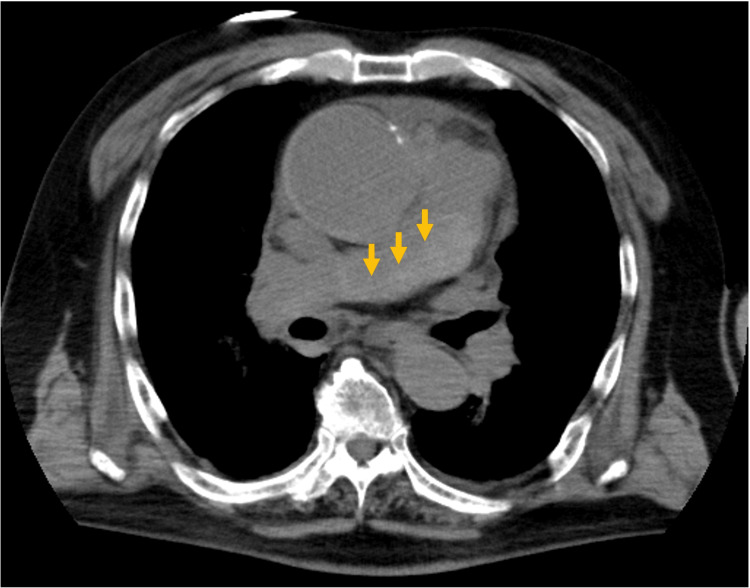
Non-contrast computed tomography of the right pulmonary artery main trunk On non-contrast computed tomography, a high-attenuation area is observed along the wall of the right pulmonary artery (yellow arrow).

Although differentiation from PTE is essential, specific findings, such as central PA stenosis and peripheral wall thickening, as seen in this case, are not typical of PTE and may aid in distinguishing the two conditions. High-attenuation areas along the PA wall on non-contrast CT may further support a diagnosis of PA-IMH [7]. This finding was also observed in this case and helped differentiate it from PTE (Figure 5).

Second, some patients with PA-IMH may present with alveolar hemorrhage. Rupture of the PA due to a hematoma extension into the peripheral PA can cause alveolar hemorrhage [8]. In such cases, respiratory failure from impaired gas exchange and excessive bleeding from aortic rupture are expected. Given the high incidence of mediastinal hemorrhage and cardiac tamponade, the condition is considered fatal. A prior study of patients with AAD and PA-IMH reported a 20% mortality rate without alveolar hemorrhage and 60% with alveolar hemorrhage. Thus, alveolar hemorrhage is recognized as an independent prognostic factor [3]. 

## Conclusions

We report a case of Stanford type A AAD complicated by PA-IMH, which is not a rare complication and may serve as an important prognostic indicator. Therefore, it is essential to identify PA-IMH using both non-contrast and contrast-enhanced CT. Some cases of PA-IMH are further complicated by alveolar hemorrhage, a poor prognostic factor that should be confirmed using CT with lung window settings.
